# Deadly Ebola virus outbreak in Uganda, 2022: An imminent threat to the public health and safety

**DOI:** 10.1097/MS9.0000000000000216

**Published:** 2023-02-07

**Authors:** Aroma Naeem, Zaofashan Zaheer, Tuaseen Kalsoom, Shehroze Tabassum, Khaled Albakri, Andrew A. Wireko

**Affiliations:** a King Edward Medical University, Lahore, Pakistan; b The Hashemite University, Zarqa, Jordan; c Sumy State University, Sumy, Ukraine

*Dear Editor*,

Sudan ebolavirus (SEBOV) is a member of the genus *Ebolavirus* (family: Filoviridae). Other five species include Zaire ebolavirus (ZEBOV); Taï Forest ebolavirus (CIEBOV), Reston ebolavirus (REBOV), *Bundibugyo ebolavirus* (BEBOV), and *Bombali ebolavirus* 1. The Ebola virus disease (EVD) is a severe hemorrhagic fever, that affects humans and nonhuman primates^1^. In most cases, an outbreak begins with a single incidence of human-to-human transmission and spreads throughout the community by direct contact with contaminated objects or body fluids. The incubation period ranges from 2 to 21 days; and the symptoms constitute fever, headache, pains, and aches in different parts including joints and muscles, and gastrointestinal signs (diarrhea, vomiting, abdominal pain, etc.), eventually resulting in a multiple organ dysfunction syndrome and often leading to death^2^. The typical EVD fatality rate is almost 50%, with ranges from 25 to 90%. Clinically, it is challenging to distinguish it from other infectious diseases, but a variety of diagnostic tests, such as the reverse-transcriptase PCR assay, the antibody-capture enzyme-linked immunosorbent assay, and virus isolation by cell culture, can provide a conclusive diagnosis. White blood cell and platelet counts are low, and liver enzyme levels are increased in the blood on laboratory testing. There is currently no approved vaccine available against the EVD-Sudan strain, hence supportive care is mainly followed for the treatment of patients^3^. Two epidemics that occurred back-to-back in several regions of Central Africa in 1976 led to the identification of the Ebola virus. The Ebola River, which gave the virus its name, is located close to where the first outbreak happened in the Democratic Republic of the Congo (formerly Zaire). About 500 miles away in South Sudan, a second epidemic occurred. Since then, the virus has occasionally infected people, causing outbreaks in numerous African nations^4^. Over 11 000 deaths have been attributed to this deadly virus worldwide to date, according to estimates^5^.

The current Ebola epidemic was officially proclaimed in Uganda on 20 September 2022, when a case of the Sudan virus disease was recorded in the Mubende area, located in the country’s center, according to a statement from the WHO. Uganda Virus Research Institute (UVRI) confirmed it after analyzing the sample, taken from a 24-year-old man who later passed away. Six deaths that occurred in his family during the same month in the same district were later confirmed by the National Rapid Response Team (NRRT). Although Sudan virus disease infections have been detected in five districts – Kyegegwa, Kagadi, Kassanda, Mubende, and Bunyangabu – the center of the disease remains to be Mubende and the district in its neighborhood, Kassanda^6^. Uganda has previously experienced four outbreaks of the same strain, the deadliest of which occurred in 2000^7^. As of 6 October 2022, 64 cases of the Ebola virus infection have been reported, with 28 deaths. Ten of the infected individuals are healthcare workers, of whom four died. Seven of the infected individuals have recovered^8^. To combat this deadly enemy immediately, a complete lockdown for 21 days has been imposed in the two most affected districts, Mubende and Kassanda^9^. The WHO is also deploying more experts, supplies, and resources to help address the new challenges, and has released two million dollars from its Contingency Fund for Emergencies (CFE)^10^. Figure 1 shows the total number of cases and deaths during previous outbreaks of Sudan strain EVD^7^,^8^.

**Figure 1:**
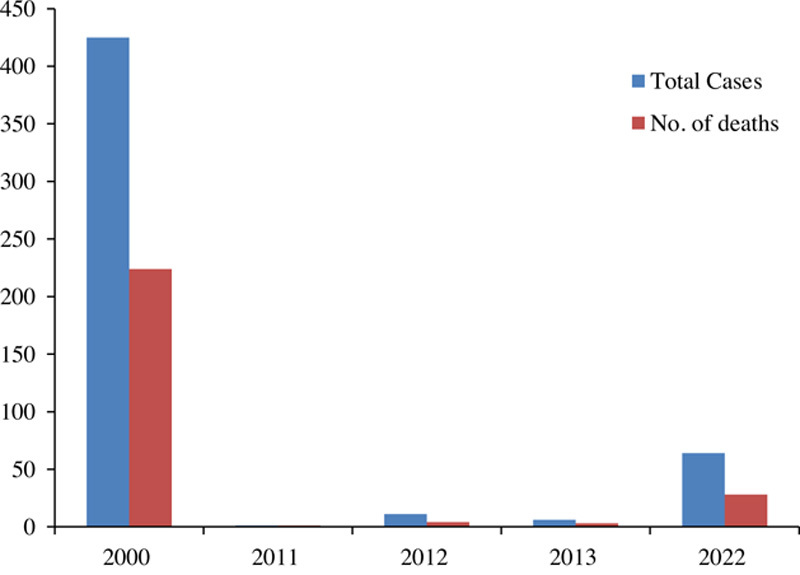
Total cases and deaths of previous Sudan strain Ebola virus outbreaks.

The recent outbreak of the Sudan Strain EVD constitutes a health crisis deserving immediate attention. While healthcare workers are in a race against time to further contain the spread of the disease, there is no preexisting vaccine for the current EVD strain. Owing to the rarity of past outbreaks, the management of Sudan strain EVD has been solely symptomatic and aimed at maintaining the homeostasis of the infected individual.^11^ Three vaccines are currently in the early stages of testing including a bivalent adenovirus vectored vaccine made by the University of Oxford and the Jenner Institute in England (ChAdOx1 biEBOV), a monovalent adenovirus vectored vaccine developed by the Sabin Vaccine Institute (ChAd3-SUDV), and a monovalent vaccine from the International AIDS Vaccine Initiative against SUDV. Although five of the 13 candidates for the EBOV vaccine have moved on to post-phase I clinical trials, there are still significant uncertainties regarding drug interactions, the safety provided by these medications, their level of effectiveness in special communities, and the potential for their long-term storage. The creation of vaccines that provide protection against all *Ebolavirus* species that are medically relevant should be the main focus of future research^12^. In addition, the entire burden of outbreak control cannot be placed on the invention of vaccines alone. In the event of an efficacious emergent vaccine, rapid large-scale production and dissemination are bound to pose a challenge for the COVID-19-ravaged health system and economy of Uganda, now also facing an outbreak of Crimean-Congo hemorrhagic fever (CCHF)^13^. The drugs currently available against the disease like Inmazeb (atoltivimab, maftivimab, and odesivimab-ebgn) target only the Zaire strain of Ebola virus. Treatment for the Sudan strain is primarily supportive and enhances the survival rates provided treatment onset is prompt. It consists of intravenous or peroral administration of fluids and electrolytes, symptomatic management, and treatment of other infections that may be contacted during the disease period^14^,^15^. Ebola virus is a communicable disease; and illiteracy, community noncompliance, reluctance with regard to standard operating procedures, unfounded fear, avoidance of hospitals and healthcare facilities, misinformation, and the mistrust bred by the rapidly spreading conspiracy theories have facilitated the outbreak. The lives of healthcare workers are especially at risk, four of whom have succumbed to the infection so far as stated above. The hospitals are not well equipped to deal with the outbreak, and the lack of basic equipment, including personal protection, is adding to the death toll. There is currently one operational Ebola Treatment Unit (ETU) in Mubende District and a lack of ETU in the remaining districts with confirmed cases. Understaffing at hospitals, lack of preparedness to deal with an outbreak, and the limited capacity of isolation units all make the situation bleak^8^. The Ebola virus tends to persist in breast milk, and other pregnancy-related fluids and tissues of women who have recovered from the disease, posing a risk of transmission to babies and healthcare workers providing obstetric and gynecological care. Moreover, the similarity between EVD and other conditions and diseases including malaria, typhoid fever, meningitis, and pregnancy symptoms has hindered rapid diagnosis and treatment^3^. Lack of resources for safe and dignified burial practices adds up to the already burning fire.

Since EBD is transmitted either via direct contact with blood and bodily secretions of the infected individual or objects contaminated with bodily secretions; disposal of needles and syringes, and careful handling and disposal of specimens from suspected individuals must be ensured. Greater resource allocation to the research sector for the invention of a vaccine against the current strain of the virus must be prioritized to contain the ongoing outbreak and prevent one in the future. While vaccine testing is still underway, a plan of action for effective dissemination should be prepared. Both the COVID-19 pandemic and the CCHF outbreak have left the healthcare system of Uganda in shambles. National and international relief efforts and the influx of aid will prove to be extremely helpful in the restoration of the health sector. One-health approach is considered optimal for monitoring zoonotic infections, involving a collaboration between infectious disease clinicians and other healthcare workers, laboratory scientists, epidemiologists, entomologists, pharmacologists, and the media^16^. As with any outbreak, containment of the spread cannot be made possible without compliance from the general public. Electronic and print media such as television and radio broadcasts in the regional languages should be used to dismiss any misinformation concerning the virus on rational grounds. Country-wide awareness programs and campaigns to educate the general public about the various risk factors, modes of transmission, symptoms, and preventive measures for EVD should be organized. There should be consolidation of the efforts for case investigations, tracing active cases and their contacts. Awareness about safe burial practices, not involving direct contact with the body of the deceased holds significant importance in controlling the spread^17^. Regular hand washing after hospital visits and taking care of patients at home ought to be made routine practice. Healthcare workers caring for pregnant women should wear gloves and appropriate protective equipment at all times. Breastmilk should be screened before the start of breastfeeding. WHO advises the adoption of safe intercourse practices for individuals who have contracted the virus to further limit person-to-person spread. Outbreak management should be included in the curricula of all healthcare workers in Africa as Ebola outbreaks are frequent here. Since specimens for diagnosis of EVD constitute a major health hazard, especially for laboratory workers, triple packaging systems should be used to package all biological specimens and testing should be carried out under strict containment conditions. Special attention should be paid to disease surveillance and case monitoring in the areas bordering the currently infected districts. The countries bordering Uganda should remain on high alert in this regard to prevent cross-border spread. The USA has already planned on redirecting all incoming passengers from Uganda to five hospitals where they will first be screened for the Ebola virus^18^. Similar practices should be adopted by other countries as well.

## Provenance and peer review

Not commissioned, externally peer reviewed.

## Ethical approval

Not applicable.

## Sources of funding

None.

## Authors’ contribution

All authors contributed equally.

## Conflicts of interest disclosure

The authors declare that they have no financial conflict of interest with regard to the content of this report.

## Research registration unique identifying number (UIN)

Not applicable.

## Guarantor

All authors.
